# Simulation of aerosol transmission on a Boeing 737 airplane with intervention measures for COVID-19 mitigation

**DOI:** 10.1063/5.0044720

**Published:** 2021-03-16

**Authors:** Khaled Talaat, Mohamed Abuhegazy, Omar A. Mahfoze, Osman Anderoglu, Svetlana V. Poroseva

**Affiliations:** 1 Nuclear Engineering Department, University of New Mexico, Albuquerque, New Mexico 87106, USA; 2 Mechanical Engineering Department, University of New Mexico, Albuquerque, New Mexico 87106, USA; 3 Department of Aeronautics, Imperial College London, London SW7 2AZ, United Kingdom

## Abstract

Identifying economically viable intervention measures to reduce COVID-19 transmission on aircraft is of critical importance especially as new SARS-CoV2 variants emerge. Computational fluid-particle dynamic simulations are employed to investigate aerosol transmission and intervention measures on a Boeing 737 cabin zone. The present study compares aerosol transmission in three models: (a) a model at full passenger capacity (60 passengers), (b) a model at reduced capacity (40 passengers), and (c) a model at full capacity with sneeze guards/shields between passengers. Lagrangian simulations are used to model aerosol transport using particle sizes in the 1–50 *μ*m range, which spans aerosols emitted during breathing, speech, and coughing. Sneeze shields placed between passengers redirect the local air flow and transfer part of the lateral momentum of the air to longitudinal momentum. This mechanism is exploited to direct more particles to the back of the seats in front of the index patient (aerosol source) and reduce lateral transfer of aerosol particles to other passengers. It is demonstrated that using sneeze shields on full capacity flights can reduce aerosol transmission to levels below that of reduced capacity flights without sneeze shields.

## I. INTRODUCTION

In-flight transmission of COVID-19 has been the subject of extensive epidemiological research.^1–8^ Several case studies highlighted the possibility of in-flight transmission of COVID-19 as evidenced by matching viral genomes collected from infected passengers.^1–3^ Many airlines have since implemented safety measures including reducing the capacity of flights and requiring passengers to wear masks. Understanding aerosol transmission and identifying effective and economically viable measures to limit disease transmission in flights remain a pressing matter.

While multiple experimental studies have been conducted on aerosol transport on aircraft,^9–11^ computational investigations can identify overlooked measures that could potentially reduce aerosol transmission between passengers. Ventilation studies have long used computational fluid dynamics to investigate and optimize ventilation systems on aircraft to improve air quality to passengers and reduce pathogen transmission.^12–18^ The numerical solution of the Reynolds-Averaged Navier Stokes Equations (RANS) with approximate turbulence closures is commonly used to estimate the flow velocity distribution within a system and has been applied in many investigations relevant to COVID-19 transmission.^19–23^ Coupling with Lagrangian particle dynamics allows for incorporation of the effects of aerosol particle size into the simulations and enables accurate modeling of the forces that act on the aerosol particles due to drag, gravity, and Brownian motion in the case of nanosized particles.^24–27^

In the context of COVID-19, the usefulness of computational fluid dynamics is constrained by the limited information on the viral shedding rates and the infectious dose of SARS-CoV2.^11,28^ Both the viral shedding rate and the infectious dose remain statistically under-characterized despite multiple efforts.^29–32^ Studies have reported substantially different shedding rates with some patients releasing orders of magnitude more virions than others.^29^ For instance, Stadnytskyi *et al.* estimated a viral shedding rate of 1000 virions per minute of speaking, but they noted that there is a large patient to patient variation as other studies reported over 100 000 virions per minute of speaking.^29,33,34^ The infectious dose is equally under-characterized and is estimated at 300–1000 virions based on animal models and other viruses such as SARS-CoV1.^31,32,35^ Despite the absence of detailed statistical characterization of shedding rates and infectious dose and their associated probability density functions, aerosol studies can still offer relative assessments of safety to guide intervention measures.

An extensive study recently published by the United States Transportation Command (USTRANSCOM) and Air Mobility Command (AMC) conducted in vitro investigation of aerosol transport in various sections of Boeing 777–200 and 767–300 airplanes using masked and unmasked mannequins and quantified the fraction of transmitted aerosol in the 1–3 *μ*m size range.^11^ The study showed that aerosol transmission was localized and mainly affected individuals in the row of an index patient (aerosol source) followed by the rows in front and behind the patient.^11^ While ventilation on aircraft is largely optimized, there may be other ways to intervene with the air flow that can help further reduce aerosol transmission between passengers. In classrooms, Abuhegazy *et al.* identified that sneeze shields/guards can substantially reduce aerosol transmission between students separated by 2.4 m by modulating the local flow field near the source and receivers.^36^ It is unclear if similar measures would be effective on aircraft given the differences in the air flow pattern, geometry, and source-receiver separation.

Boeing 737–800 and 737–700 are the two most popular aircraft models in the U.S. fleet as of 2019. The objective of the present investigation is to study in-flight aerosol transmission and surface contamination using an in-house developed computational model of a cabin zone of Boeing 737. The cabin model contains 60 passengers spread across 10 rows of seats at full capacity and 40 passengers at reduced capacity. The investigation aims to understand the effect of reducing passenger capacity and to compare to alternative intervention measures that may be more economically viable such as using sneeze shields (sneeze guards) between passengers on a full capacity flight. The investigation considers a wide range of particle sizes (1–50 *μ*m), which spans particles released during exhalation, speech, and coughing.^37,38^

## II. METHODS

### A. Cabin model and spatial mesh

**FIG. 1.**
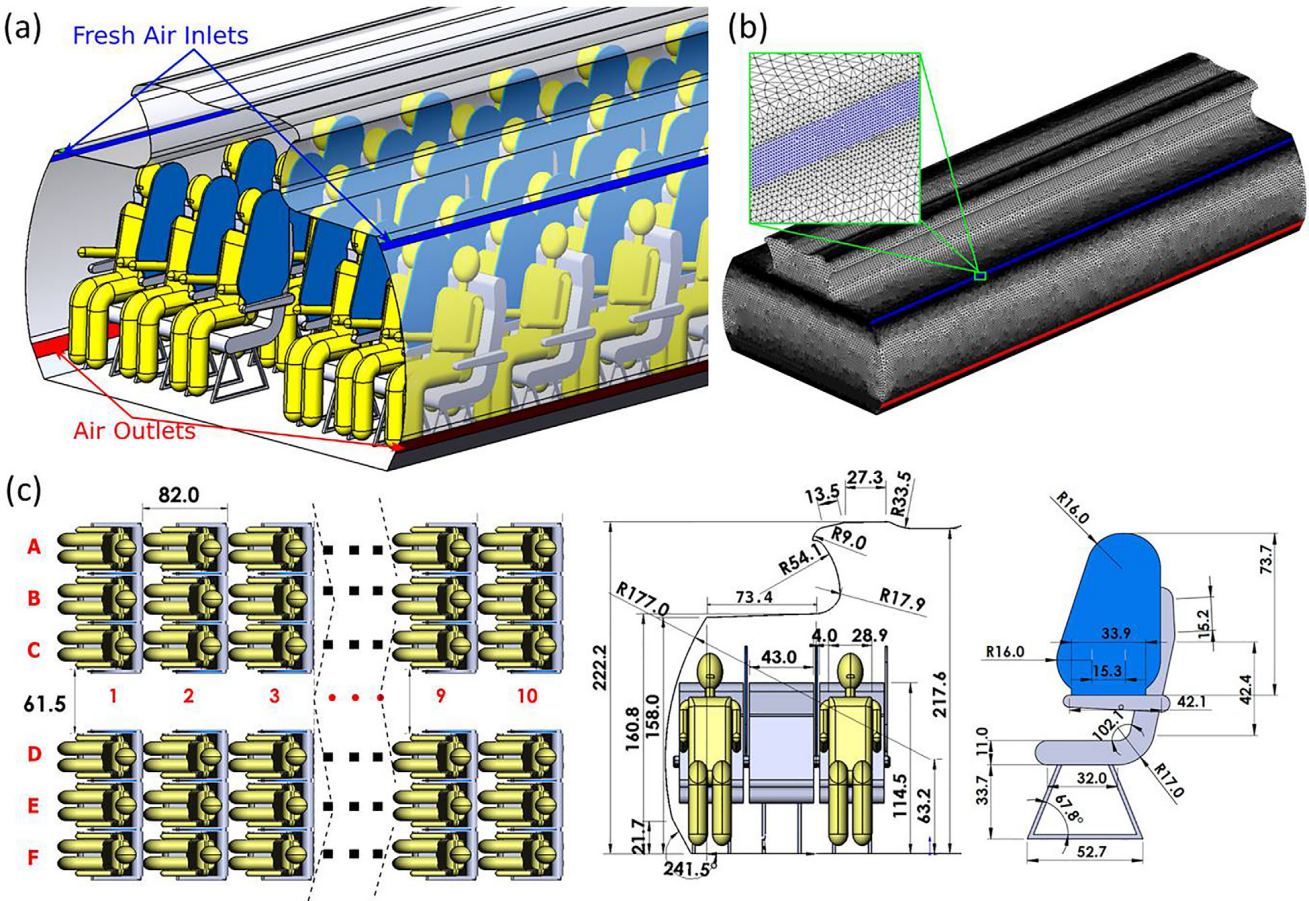
(a) Illustration of the Boeing 737 cabin model with sneeze shields/guards, (b) the computational mesh used, and (c) dimensions of the model in cm.

A three dimensional model of a Boeing 737 cabin zone was developed based on publicly available information from Boeing Commercial Airplanes released for airport planning.^39^ The dimensions of the cabin, seats, and sneeze shields/guards are shown in detail in Fig. 1. The model represents a zone containing 60 seats divided into ten rows. Passengers are assumed to be sitting upright, facing front, and in a stationary state [Fig. 1(a)]. While accurate geometry is used for the airplane walls and seats, simplified human models are used to represent the passengers in order to reduce the cost of simulations and potential sources of error and uncertainty associated with mesh generation for the simulations, as done in other studies.^40,41^ The passengers are assumed to be ∼138 cm in height when seated and ∼37 cm in width [Fig. 1(c)]. Shields placed between passengers are 2 cm in thickness and ∼74 cm in height and cover the length of the armrest of the seats.

Ventilation of the cabin model follows ASHRAE Standard 161–2018 for air quality in commercial aircraft.^42^ Per the standard, a minimum air supply of 15 cubic feet per minute (CFM) per person is required, and 20 CFM per person is recommended.^42^ Therefore, the ventilation system in the model was assumed to supply 1200 CFM (566 l/s) of air as there are up to 60 passengers in the cabin zone. The supply and exhaust diffusers of the ventilation system are shown in Fig. 1 with a configuration similar to that in the study by Liu *et al.*.^43^ A velocity inlet boundary condition is specified with 0.65 m/s in the vertical direction and 1.13 m/s in the transverse direction, which gives 1200 CFM at 30° angle with the tangent to the wall body of the airplane.^43^ Given that air in commercial aircraft is typically pressurized to the equivalent to outside air pressure at ∼2400 m of elevation, the exhaust diffusers are assumed to be at a similar pressure of 75 kPa.

A highly fine unstructured tetrahedral mesh is used with near-wall refinement [Fig. 1(b)]. The mesh was generated using ANSYS ICEM 19.1. To ensure the similarity of the computational mesh between the different cases to the extent possible, only one mesh was generated for all cases. Specific cell zones are activated and deactivated as necessary to account for shields and middle passengers. The generated mesh had a maximum grid size of ∼8 cm in the aisle area away from surfaces. Much smaller grid sizes are used near surfaces where aerosol deposits or exits the system. A grid size of ∼0.25 cm is used for mouth surfaces, resulting in 155 mesh cells for each mouth. Inlets and outlets of the ventilation system were refined to ∼0.4 cm in grid size [Fig. 1(b)]. A recent study of airflow and aerosol transmission on a cabin model showed that ∼11 million elements were sufficient for mesh independence within 3%.^18^ The computational mesh used herein consists of 14.8 × 10^6^ to 19.2 × 10^6^ cells depending on the passenger capacity. The model with reduced passenger capacity has more cells than the full capacity model as the cell zones occupied by the middle passengers are activated as fluid cells. To further verify that the computations are sufficiently independent from the mesh, the results obtained for one case using the current mesh were compared against those obtained using a much finer mesh with 34× 10^6^ elements. The differences observed did not suggest the need for additional refinement of the base mesh.

### B. Numerical simulations

The simulations conducted in the present investigation follow a similar methodology as our earlier investigation on aerosol transport in classrooms and solve the same equations described.^36^ ANSYS FLUENT 19.1 is used to estimate the velocity field of the air and simulate the particle dynamics. More particularly, the numerical solution of Reynolds Averaged Navier-Stokes equations with Re-Normalization Group (RNG) k-ε turbulence closure is employed to simulate the continuum phase.^44^ The suitability of the RNG k-ε model for modeling airflows on aircraft has been previously investigated and compared to experimental results and other approaches to turbulence modeling such as large eddy simulations (LES) and detached eddy simulations (DES).^43^ The work by Liu *et al.* showed very good agreement between the velocity profiles predicted using the RNG k-ε turbulence model and experiments for an isothermal unoccupied cabin.^43^ However, the model was outperformed by more computationally expensive LES and DES in 4 of 11 sampling positions when a fully occupied cabin was considered with thermally heated mannequins and energy coupling.^43^ Nevertheless, the data they reported indicate the suitability of the RNG k-ε model for the present application with some trade-off between accuracy and computational cost compared to LES and DES.

As the concentration of the aerosol particles relevant to the present application is too small to appreciably affect the continuum phase (air), the continuum phase was simulated first independent of the aerosol particles. Second-order finite volume discretization of the convection and viscous terms of the RANS equations was used. The SIMPLE algorithm with first-order pressure interpolation was employed for pressure-velocity coupling. No-slip boundary condition is applied to walls, and inlet/outlet boundary conditions are defined per the ventilation requirements. All passengers are assumed to inhale air at 20 l/min except the aerosol source [passenger 7C in Fig. 1(c)], who exhales at 20 l/min. Each simulation was run for 5000–8000 iterations depending on the mesh size until the convergence of the residuals to a maximum of 10^−4^ for k, ε, x, y, and z momentum equations and the mass continuity equation. The continuum phase simulations employed time-independent boundary conditions. The continuum phase solver was, then, frozen, and the converged velocity field was used as input to the particle dynamics solver.

The aerosol particles were modeled as a discrete phase under a Lagrangian tracking framework. The Lagrangian approach allows for direct incorporation of the effects of particle size on drag and gravitational forces on the particle.^25^ However, it necessitates the simulation of a large number of particles to obtain aerosol distributions sufficiently independent of the particle count.^25,45^ The present simulations used 300,000 particles each. It is well known that large outlier particles can skew mass-based deposition distributions in polydisperse particle simulations due to the cubic scaling of particle mass with diameter. Therefore, independent monodisperse simulations were carried out for different particle sizes. The fate of exhaled particles is determined by solving the equation of motion for each particle while employing the Stokes-Cunningham drag model.^46^ The use of a drag model is necessary as the particles are too small to be directly resolved by mesh elements within the simulated system. As momentum diffusivity of air is the dominant mode of particle transport, the particles were approximated as spherical particles. Notably, such a shape approximation may not be applicable when thermal motion is the dominant mode of transport as in the case of transport of a virus within highly viscous fluids such as cells.^47^ In such a situation, accounting for an accurate shape and rotational diffusivity of the virus particle becomes necessary to model thermal motion and particle orientation.^47^ Particles are assumed to be trapped upon impact with surfaces. This assumption follows from the fact that micrometer sized particles can effectively attach to surfaces through van der Waals forces. The adhesion forces acting on 1 μm particles, for instance, can exceed gravitational forces by six orders of magnitude.^48^ As monodisperse simulations are performed, evaporation of larger particles upon impact with surfaces and disintegration into smaller particles that reenter the air is not considered.

### C. Study design

The separate continuum phase and Lagrangian simulations are conducted for each of the three models: (a) model at full passenger capacity (60 passengers), (b) model with reduced passenger capacity (40 passengers), and (c) model at full passenger capacity with sneeze guards/shields between passengers. One passenger located on the seventh row [7C seat in Fig. 1(c)] of the cabin zone exhales aerosol particles, which may be inhaled by other passengers. The index patient is the only source of aerosol particles in the simulations. The standard filtration efficiency of particulate filters used in the aerospace industry exceeds 99.99%.^49^ Aerosol particles that exit through the outlet are assumed to be perfectly filtered and are not recycled into the system. Susceptible passengers are assumed to inhale air at a rate of 20 l/min and can, therefore, inhale aerosol particles in the air. The inhalable aerosol fraction and the fraction of aerosol deposited on various surfaces (including passengers) are quantified for relative risk assessment. Inhalation and exhalation cycles are not considered as steady state continuum phase simulations are employed. The inhalable fraction is uncorrected for the inhalation-to-exhalation time ratio and internal deposition fraction. The inhalable fractions reported herein are, therefore, useful for relative comparison of the intervention measures and do not represent absolute values for inhalation dosimetry. A wide range of particle sizes (1–50 *μ*m) is investigated for each of the three models considered to understand the effect of particle size on the efficacy of the measures in reducing aerosol transfer and passenger contamination.

## III. RESULTS AND DISCUSSION

### A. Airflow in the cabin

**FIG. 2.**
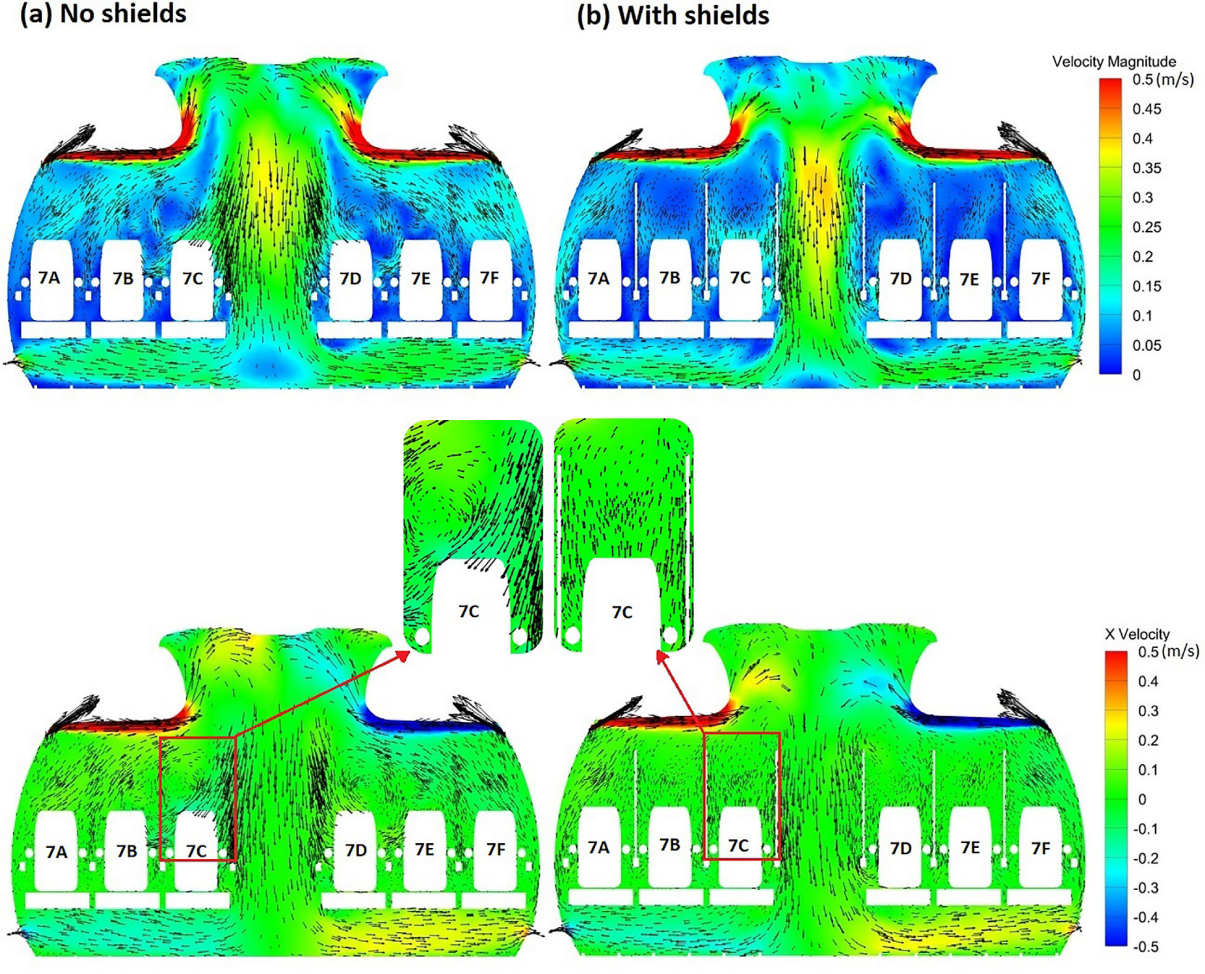
Distribution of the velocity magnitude and lateral velocity component through a section of the cabin for (a) the model with no shields and (b) the model with shields.

Air flow in the cabin is mainly controlled by the ventilation system and the geometry of objects and walls inside the cabin. The velocity vectors in Fig. 2 show that the flow circulates tangential to the cabin walls in the upward direction and moves downward in the aisle and then flows horizontally to the outlets. In the passenger area, the flow circulates with tendency for lateral flow. The velocity magnitude is the strongest near the inlet followed by the aisle and the area near the outlet toward the bottom of the plane. It can be observed that the distribution of air velocity in the cabin is largely symmetric. The velocity magnitude exhibits weak asymmetry near the top of the plane in the flow separation region. Weak asymmetry in the velocity magnitude of the air can arise from the presence of an exhaling passenger on the right side of the plane (passenger 7C) and the longitudinal asymmetry in seat offset distance from the front and back walls of the cabin. The lateral component of velocity (x-velocity) exhibits stronger symmetry than the velocity magnitude. In a real cabin, experimental studies have shown that the flow tends to be more asymmetric due to differences in passenger size and heat generation.^50,51^ The present work, however, is concerned with an idealized scenario, which produces a mostly symmetric field.

**FIG. 3.**
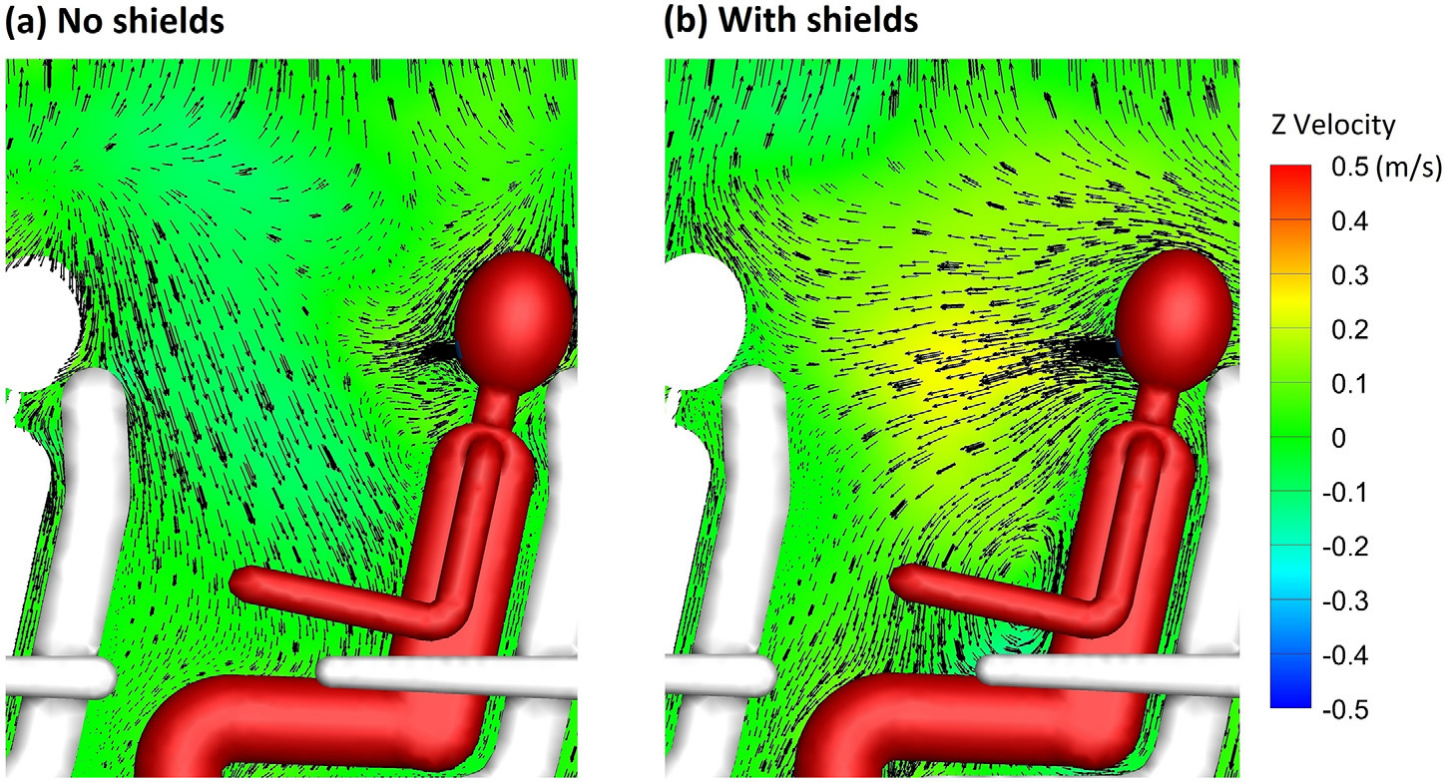
Comparison of longitudinal velocity in (a) the model with no shields and (b) the model with shields (hidden).

A comparison of the velocity distribution of air through a section of the cabin in proximity to the index patient reveals substantial differences in the flow pattern after the inclusion of sneeze shields/guards in the full passenger capacity model [Figs. 2(a) and 2(b)]. In the full capacity case with no shields [Fig. 2(a)], relatively strong vortices are observed in the passenger area, leading to increased lateral flow of air in the region. The shields [Fig. 2(b)] serve to convert part of the lateral momentum to longitudinal momentum through redirecting the flow. In the case with shields [Fig. 2(b)], the velocity vectors in the passenger area have substantially weaker magnitude in the lateral direction than the case with no shields. The effect of shields on the longitudinal velocity component is illustrated through the comparison in Fig. 3. It can be observed that the longitudinal velocity component is relatively weak in the case without shields [Fig. 3(a)]. When shields are used, air is directed forward as part of the lateral momentum is converted into longitudinal momentum [Fig. 3(b)]. The longitudinal flow pattern and vortex structures in the passenger area are substantially affected by the shields. Shields also affect the global airflow in the cabin. Differences in velocity magnitude are apparent near the top of the plane (Fig. 2). The stronger currents are closer to the top of the plane in the case with no shields [Fig. 2(a)] but are shifted downward closer to the flow separation region in the case with shields [Fig. 2(b)].

Differences in the flow pattern illustrated in Figs. 2 and 3 between the model with shields and the model without shields can substantially influence aerosol and pathogen transport within the cabin. Weaker lateral currents would reduce lateral transport of aerosol particles, while stronger longitudinal currents would facilitate longitudinal aerosol transport. An increase in longitudinal aerosol transport compared to lateral transport may be desired. By increasing longitudinal aerosol transport and reducing lateral transport, more particles would be blocked by the seats. The present work is focused on comparing the efficacy of shields, which in part utilize this mechanism to reduce aerosol transmission, to the widely used intervention measure of reducing passenger capacity through vacating the middle seat. It is important to note that separate simulations are conducted for the cases with reduced passenger capacity as it has been shown in other works that individuals can influence the flow.^43^

### B. Particle dynamics and surface contamination

**FIG. 4.**
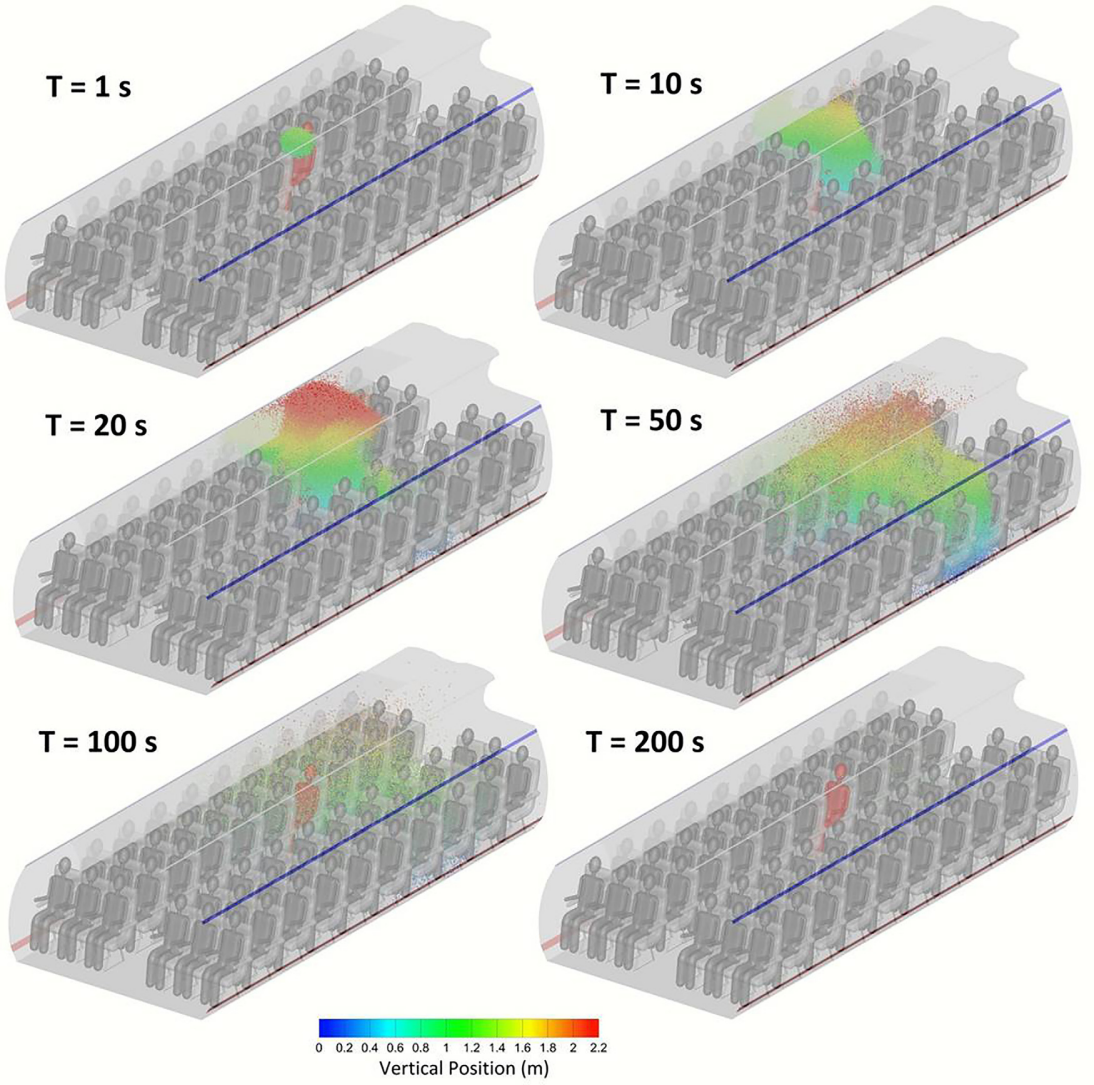
Distribution of 1 *μ*m particles in the full passenger capacity model at different points in time.

An impulse source in time was simulated in order to investigate the spatiotemporal dynamics of the aerosol particles. The three dimensional distribution of the aerosol particles at different points in time after release is shown in Fig. 4 for 1 *μ*m particles. Within only 10 s of release, the particles begin to be transmitted to other individuals. As the particles disperse in the air, some particles rise to the ceiling level after ∼20 s. Until that point, the majority of the particles were localized in the side of the index patient. Particles that rise to the ceiling level where the lateral velocity of the air is stronger (Fig. 2) tend to move rapidly to the other side of the plane. At 50 s, the concentration of particles on the other side of the plane becomes significant. The concentration of aerosol in air continuously decreases as particles disperse and deposit on surfaces or exit the cabin through the outlet diffuser. After 200 s, the vast majority of the particles had either deposited or exited the cabin. Few particles remain trapped in vortices. Most important, however, is that it can be observed from Fig. 4 that particles do not disperse through the entire cabin space at any point in time. The particles remain localized within one or two rows at most from the index patient. This suggests that the presence of one patient does not contaminate the whole cabin space.

**FIG. 5.**
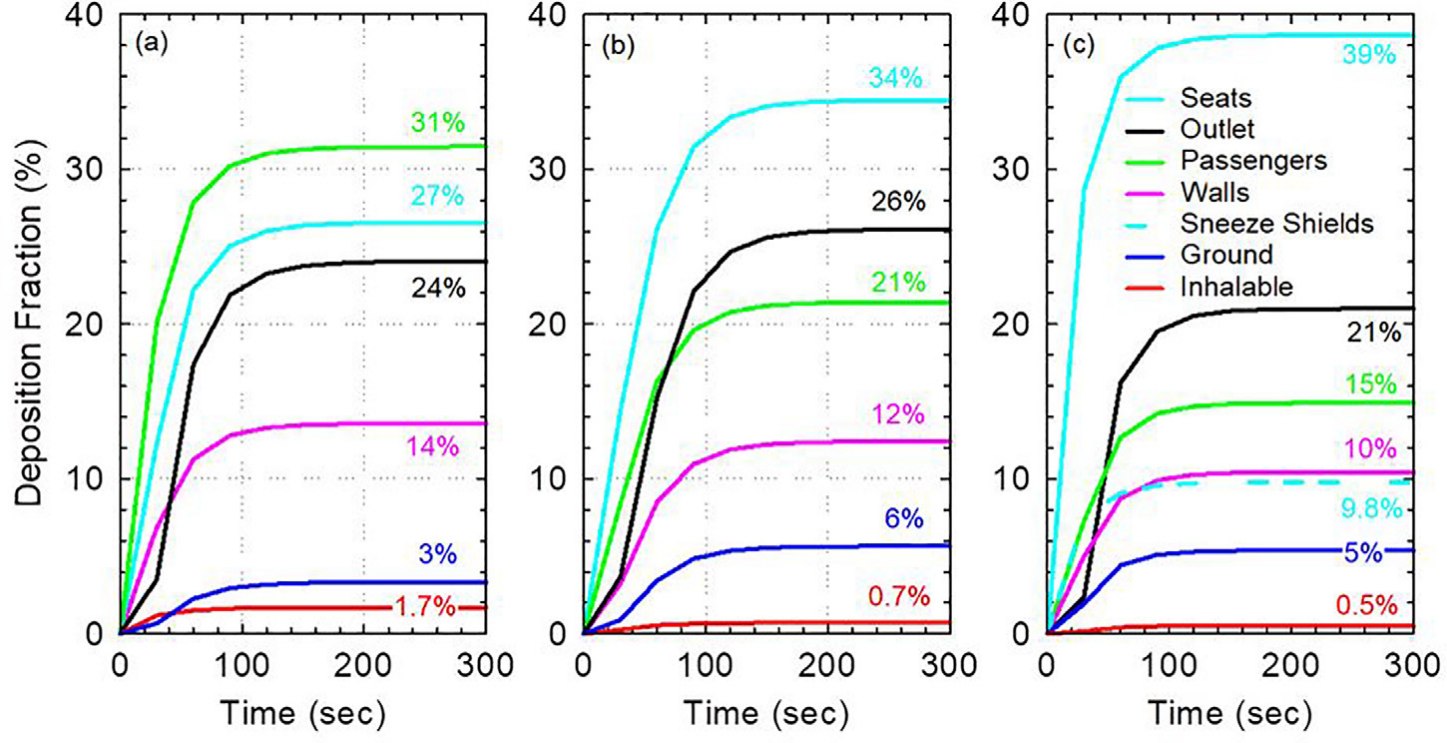
Surface contamination with 1 *μ*m particles as a function of time for (a) the full capacity model with no sneeze guards, (b) reduced capacity model with no sneeze guards, and (c) full capacity model with sneeze guards.

Direct contact with contaminated surfaces is one route for disease transmission. It is, therefore, necessary to analyze surface contamination in the cabin and study the fate of exhaled aerosol particles. Figure 5 shows the fractions of aerosol deposited on different surfaces in the cabin as a function of time in the three models considered for 1 *μ*m particles. All exhaled particles deposit on a surface or leave the system within 2–3 min [Figs. 5(a)–5(c)]. Interestingly, a relatively small fraction (21–26%) of exhaled particles are directly removed by the ventilation system. The majority of the particles deposit on surfaces in the cabin. Substantially, more 1 *μ*m particles deposit on the walls than on the ground (10–14% vs 3%–6%). This is expected due to the large surface area of the walls and the relatively weak effect of gravity on 1 μm particles compared to the effect of the flow.

Comparing different models, we found that the most contaminated surfaces in the full capacity model with no sneeze guards are the passengers (including the index patient) at 31% deposition fraction followed by the seats at 27% [Fig. 5(a)]. In the reduced capacity model with no sneeze guards and the full capacity model with sneeze guards, total deposition on passengers is reduced to 21% and 15%, respectively [Figs. 5(b) and 5(c)]. The reduction in the deposition in passengers is largely due to the increase in the deposition on the seats in both models. The increase in deposition on seats in the model with reduced capacity is due to deposition on the vacant middle seat. Meanwhile, in the full capacity model with sneeze guards, the increase in deposition on seats is due to the increased forward momentum of the flow in the longitudinal direction due to the interaction of air with the shields (Fig. 2). This is consistent with the observation that deposition on walls is reduced from 14% to 10% as the lateral flow becomes weaker. Furthermore, a fraction of the exhaled 1 *μ*m particles (9.8%) deposit directly on the sneeze guards. The deposition on the shields (9.8%) is equivalent to 46% of those directly removed by the ventilation system and is, therefore, quite significant.

The total inhalable fraction is the lowest in the full capacity model with sneeze guards (0.5%) followed by the reduced passenger capacity model without sneeze guards (0.7%) and then the full capacity model without sneeze guards (1.7%). As the passengers are assumed to be continuously inhaling since steady state simulations are employed for the continuum phase, this inhalable fraction quantity is useful for relative comparison of the models and not for inhalation dosimetry. It overestimates the fraction of particles that would be inhaled in reality as the passengers are not assumed to exhale particles back and are assumed to be continuously inhaling air at all times. Adjusted estimates for comparison with experimental data may be obtained by multiplying the inhalable fraction by the deposition fraction of the particles in the human respiratory system and by the ratio of time spent inhaling to the time spent exhaling in real passengers.

**FIG. 6.**
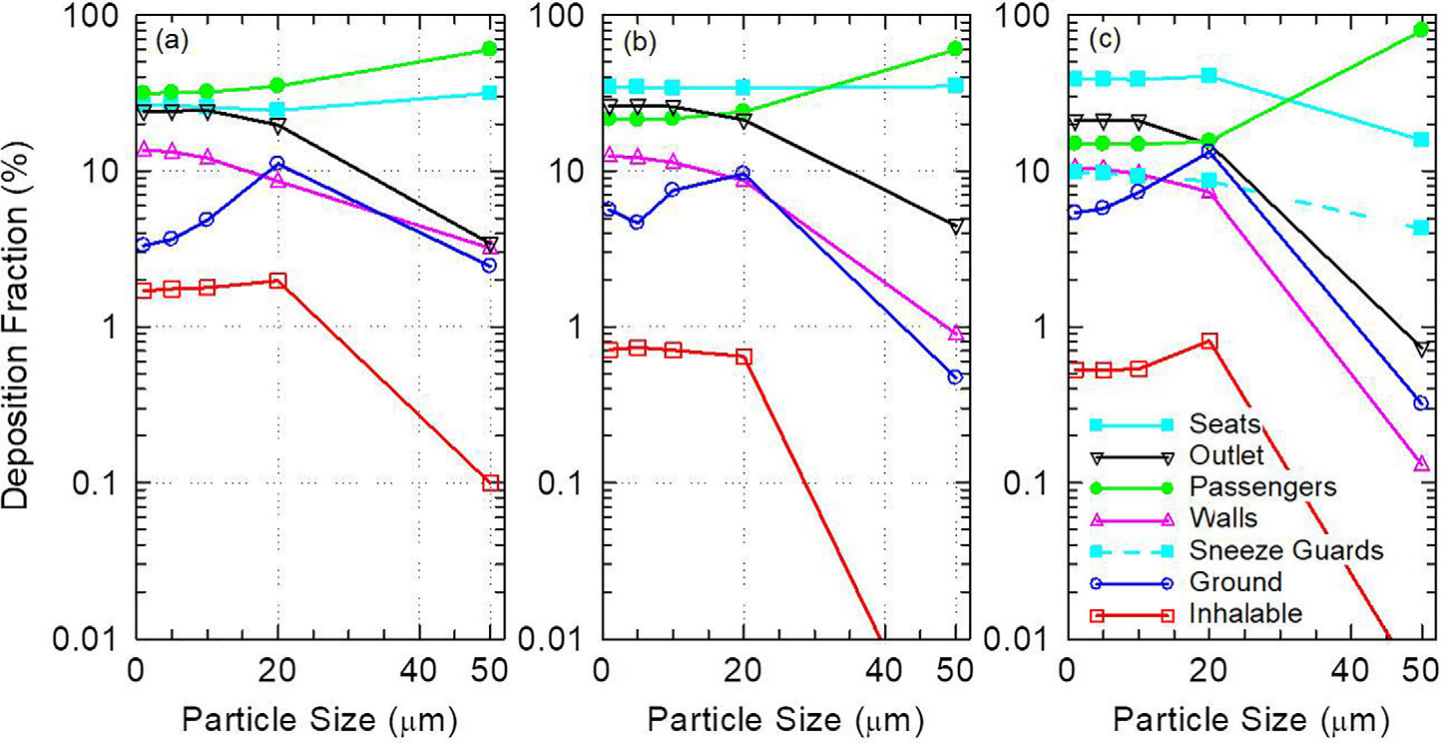
Surface contamination as a function of particle size for (a) the full capacity model with no sneeze guards, (b) the reduced capacity model with no sneeze guards, and (c) the full capacity model with sneeze guards. Values below 0.01% are not shown.

The distribution of aerosol deposition in the cabin depends on particle size as shown in Fig. 6. Aerosol deposition on passengers increases with increased particle size especially for particles ≥ 10 *μ*m. This is expected as the relative influence of gravitational force to aerodynamic drag on the particles increases with increased particle size, which leads to increased deposition on the source passenger and nearby passengers [Figs. 6(a)–6(c)]. Similarly, aerosol deposition on walls decreases consistently with increased particle size as the mean distance traveled by the particles from the source decreases [Figs. 6(a)–6(c)]. Interestingly, sneeze guards are more effective at directly stopping smaller particles than they are at stopping larger particles [Fig. 6(c)]. About 8.6% of 20 *μ*m particles deposit directly on sneeze guards compared to 9.8% of 1 μm particles. Meanwhile, only 4.3% of 50 *μ*m particles deposit on the shields. This can be explained by the downward favoring trajectory of 50 *μ*m particles, which reduces the chances of direct intersection of their trajectories with the shields. Deposition on the ground also tends to increase with increased particle size except for 50 μm particles, which tend to deposit on the passengers before they can make it to the ground.

Reduction in passenger capacity and use of sneeze guards eliminates the direct transmission of 50 *μ*m particles. Although 50 *μ*m particles deliver a much smaller inhalable fraction compared to smaller particles such as 1 *μ*m particles (Fig. 6), they contain substantially more virions than 1 μm particles due to their volume.^30^ The inhalable aerosol fraction of 50 *μ*m particles (0.1%) is only 17 times less than that of 1 *μ*m particles despite having 125 000 times the mass [Fig. 6(a)]. This suggests that large particles such as 50 *μ*m particles, which can be released during coughing and sneezing, carry much higher risk for short-range transmission than smaller particles released during exhalation and talking. Larger particles, however, carry less risk for long-range transmission due to their short mean free path. It is, therefore, quite significant that measures such as reduction in passenger capacity and the use of sneeze guards can practically eliminate the short-range transmission of 50 *μ*m particles.

### C. Aerosol transmission to passengers

**FIG. 7.**
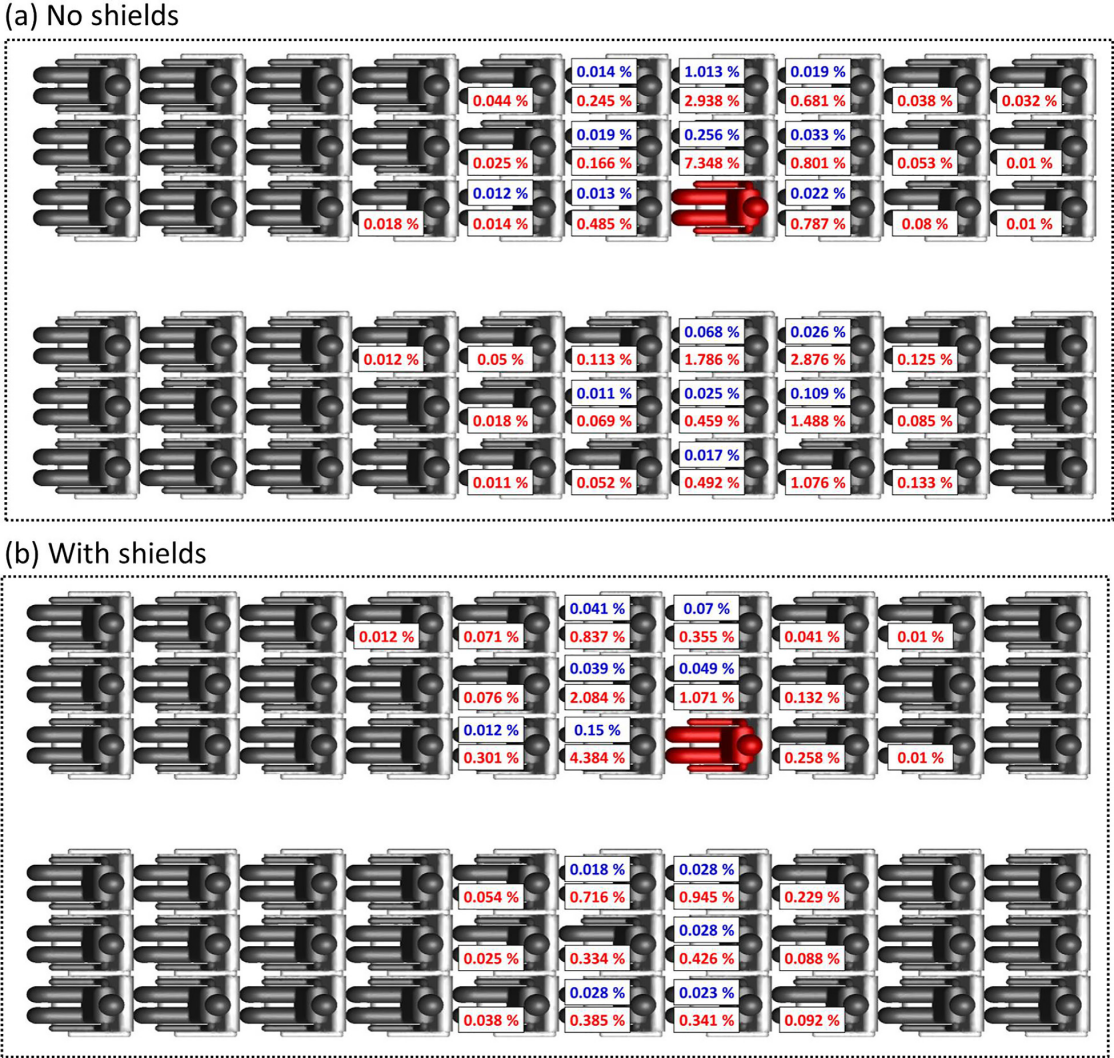
Distribution of the inhalable fraction (blue) and deposition fraction (red) of 1 μm particles among passengers in the full capacity models: (a) with no sneeze shields/guards and (b) with sneeze shields/guards. A threshold of 0.01% is applied to data.

The distribution of the deposition fraction (red) and inhalable fraction (blue) among passengers is shown in Fig. 7 for 1 *μ*m aerosol particles in the full passenger capacity cases. A threshold of 0.01%, which corresponds to 30 particles, is applied to the data. Significant lateral transfer of particles is observed relative to longitudinal transfer in the model with no shields/sneeze guards [Fig. 7(a)]. Individuals in the same row of the index patient receive the most particles with one of the passengers inhaling up to ∼1% of the released particles. Particles are transmitted to passengers on both sides of the plane and not only the side of the index patient (highlighted in red). It can be observed from Fig. 7 that there is no consistent relation between the fraction of aerosol inhaled and deposited on passengers. This is expected for three reasons: (a) the velocity field of the air near passengers exhibits strong vorticity (Fig. 2), (b) different passengers are oriented differently with respect to the source, and (c) air flow is affected by passengers and objects present between the source and other passengers.

A significant increase in the fraction of particles delivered to passengers in the row immediately in front of the index patient is observed when shields/sneeze guards are used. Nevertheless, overall, there is a 70% reduction in the inhalable fraction and 42% reduction in the total deposition fraction to passengers due to substantial reduction in lateral particle transfer when shields are used [Fig. 7(b)]. The increase in the fraction of particles delivered to passengers in front of the index patient is expected as the shields direct the flow forward. Pushing the flow forward is the main mechanism through which shields reduce the overall particle transfer to passengers as a fraction of the particles pushed forward deposit on the back of the seats in front of the index patient. Aerosol transmission to individual passengers is dependent on the transport of the aerosol particles in vortices present in the passenger area (Fig. 2). Vortices in the passenger area are affected by model assumptions such as the turbulence model used, simplified passenger geometry, stationarity of passengers, and isothermality. Therefore, the amount of aerosol transmitted to individual passengers cannot be ascertained. Nevertheless, the data in Fig. 7 clearly show that particles are concentrated within one row from the index patient in the model without shields and the model with shields. Individuals within one row of the index patient receive orders of magnitude more particles than passengers located in farther rows. Virtually, no particles make it past two rows from the index patient.

**FIG. 8.**
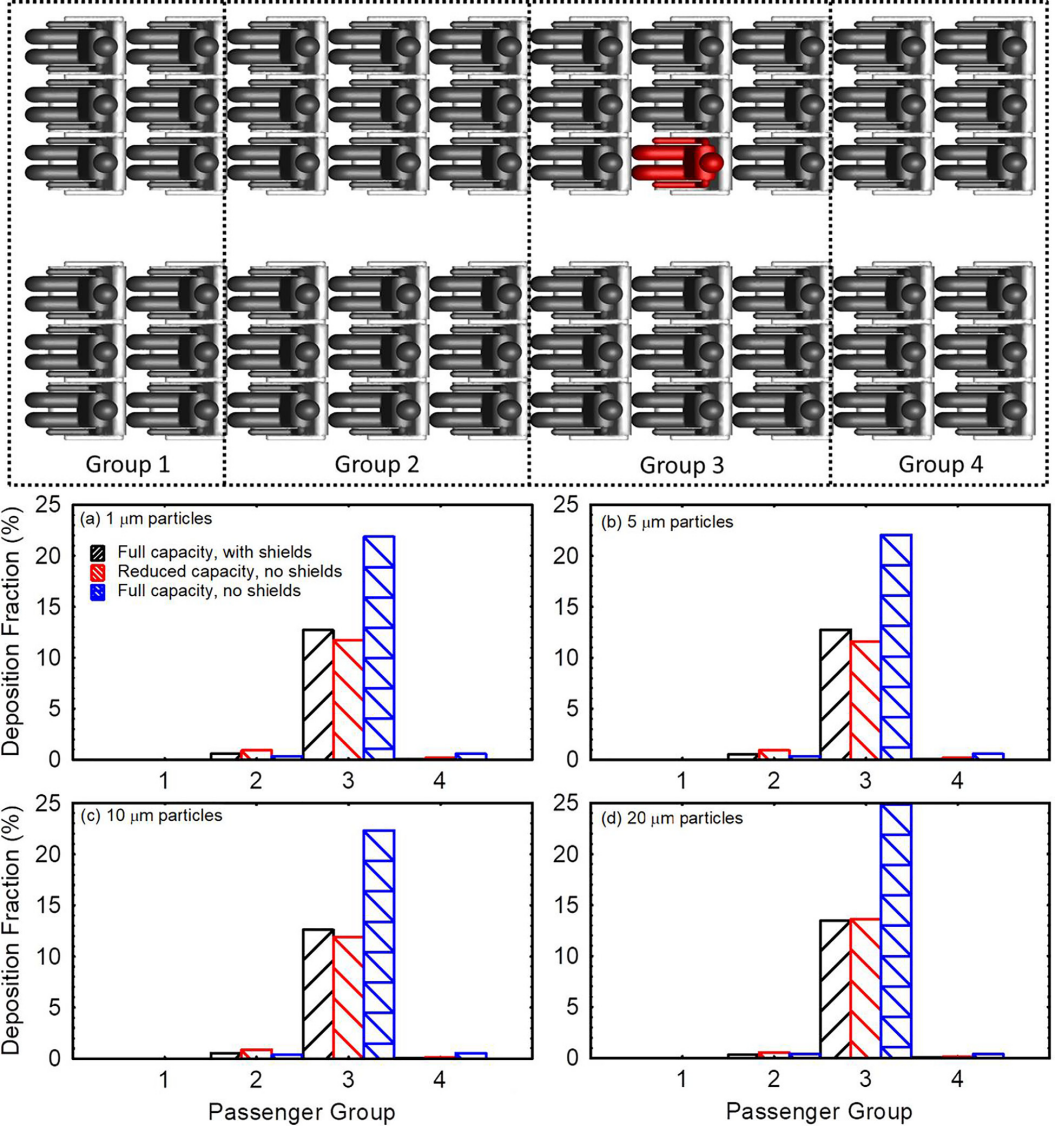
Comparison of the aggregate deposition fraction delivered to different passenger groups in the three models for (a) 1 *μ*m particles, (b) 5 *μ*m particles, (c) 10 *μ*m particles, and (d) 20 *μ*m particles.

Due to uncertainties in aerosol transmission at the individual passenger level, the fraction of aerosol transmitted to individual passengers through deposition or direct inhalation (Fig. 7) is not immediately useful for comparison of the efficacy of different intervention measures. It is more useful when comparing different intervention measures to divide passengers into groups and tally the deposited and inhaled fractions within each group. In Fig. 8, the passengers are divided into four groups by the location. The first and last groups consist of two rows of passengers each. The two middle groups consist of three rows of passengers each. The source passenger is located in the middle of group 3 (highlighted in red). Deposition on the source passenger is excluded from group 3 deposition tallies.

Figures 8(a)–8(d) present the fraction of particles deposited on passengers in each group for 1, 5, 10, and 20 *μ*m particles, respectively. Group 3 passengers are at the highest risk followed by group 4 passengers for the full capacity model with no shields. Passengers in group 3 receive in aggregate 37, 38, 43, and 65 times the aerosol particles as group 4 passengers for 1, 5, 10, and 20 μm particles, respectively. In the case with shields, group 3 passengers remain at the highest risk followed by group 2 passengers. Introducing shields to a full capacity flight reduces particle deposition on passengers in aggregate by 41%–45% depending on particle size. Meanwhile, reducing passenger capacity to 40 passengers through vacating the middle seats reduces the total fraction of aerosol particles deposited on passengers (excluding source) by ∼45% compared to the full capacity case without shields. This 45% reduction in aggregate deposition is mostly due to the reduced number of passengers and partially because removed individuals in proximity of the source received a greater amount of particles.

**FIG. 9.**
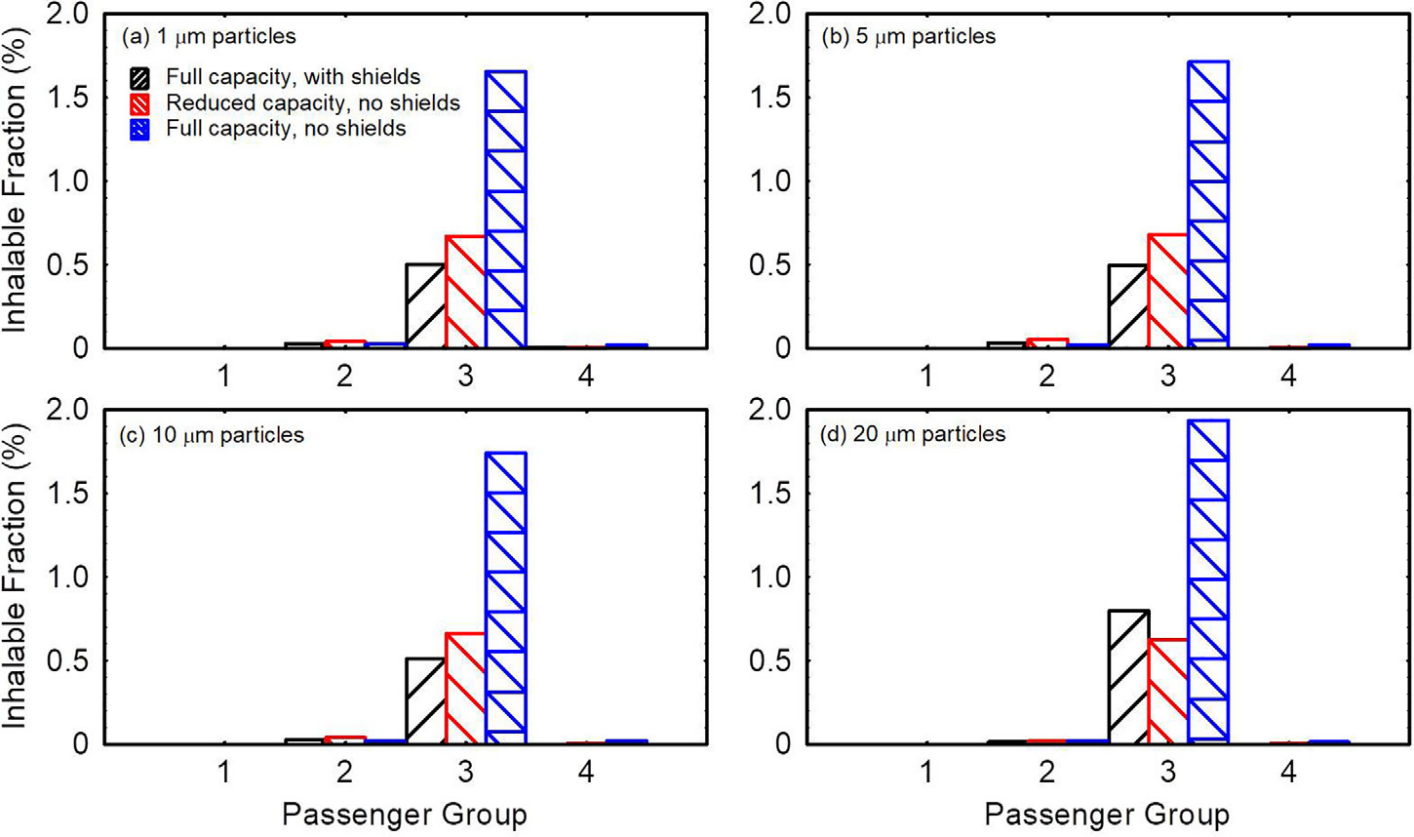
Comparison of the aggregate inhalable fraction delivered to different passenger groups in the three models for (a) 1 *μ*m particles, (b) 5 *μ*m particles, (c) 10 *μ*m particles, and (d) 20 *μ*m particles.

Figure 9 compares the total inhalable fraction delivered to different groups in the three models considered. Shields/Sneeze guards reduce the inhalable fraction for 1–10 *μ*m particles by 68–70% and by ∼59% for 20 *μ*m particles in the full capacity model [Figs. 9(a)–9(d)]. Vacating the middle passenger seats, on the other hand, reduces the total inhalable fraction by 57%–60% for 1–10 μm particles and by 67% for 20 *μ*m particles compared to the full capacity model with no shields [Figs. 9(a)–9(d)]. Therefore, it is clear from Figs. 8 and 9 that reducing the passenger capacity and vacating the middle seats result in nearly equivalent reduction in particle transfer in aggregate. The rationale behind focusing the comparisons in Figs. 8 and 9 on aggregate values rather than average values per person is that the risk of someone being sick is proportional to the number of passengers on the flight. The aggregate values are equivalent to multiplying the average values per person by the number of people on the flight. Therefore, aggregate values offer indicators of risk, which relatively account for the number of potential patients on the flight. Per-person values can be directly derived by dividing the data in Figs. 8 and 9 by the number of passengers in each group.

Although the total deposition on passengers in the reduced capacity model is slightly lower than the full capacity model with shields, the average deposition per passenger is 30%–35% lower in the full capacity model with shields. This indicates a stronger effect of shields on aerosol transmission than vacating middle seats. Compared to the full capacity flight with no shields, vacating the middle seat reduces the average aerosol deposition per passenger in the 1–20 μm size range by 15–17% and reduces the average amount of aerosol inhaled per passenger by 36–51%. This is less effective than shields, which reduce deposition per passenger by 41%–45% and reduce the inhalable fraction by 59–70%. Nevertheless, per-passenger values are only useful if the patient can be identified. In case the patient is unknown, aggregate values provide a better indicator of risk.

## IV. CONCLUSIONS AND RECOMMENDATIONS

The present work compared different intervention measures to reduce aerosol transmission on an aircraft cabin using computational fluid particle dynamics. Three models were developed for a Boeing 737 cabin zone: (a) model with 60 passengers (full capacity), (b) model with 40 passengers (reduced capacity), and (c) model with 60 passengers with sneeze guards between the passengers. In addition to directly blocking a fraction of the aerosol particles, the sneeze guards were designed (Fig. 1) such that they converted part of the lateral momentum of air into longitudinal momentum (Fig. 2) to increase aerosol deposition on seats and reduce aerosol transmission to passengers. Two measures were used to compare aerosol transmission to passengers: (a) aggregate inhalable fraction (uncorrected for internal deposition and exhalation) and (b) aggregate deposition fraction on passengers. The study considered a wide range of particle sizes from 1 *μ*m to 50 *μ*m.

The present study is subject to several assumptions and limitations. First, all passengers are assumed to be of the same height and size. It is well understood that passengers can influence the flow, and thus, variability in height and size can affect the flow distribution in the cabin.^43^ Second, passengers are assumed to be stationary and facing forward at all times. Changes in the head orientation could affect the aerosol distribution in the cabin especially for larger particles ≥ 20 *μ*m. Motion of passengers in the aisle or otherwise is also not considered. Third, the plane is assumed to be cruising at a constant velocity and no tilt. Tilt of the airplane and acceleration may slightly affect the particle distribution. Fourth, the inhalable fractions calculated are intended for relative comparison of the models but not for dosimetry purposes. For dosimetry purposes, it is necessary to consider nasal breathing with accurate nose shape and study the internal deposition of the aerosol particles and the factors that affect deposition such as head orientation as a fraction of the particles are exhaled back.^28,52,53^ Fifth, the present study assumes rigid and nonporous sneeze guards with identical size and shape. It is unclear how using nonrigid sneeze guards would affect the present findings. Sixth, only one cabin zone of a widely used commercial airplane (Boeing 737) was modeled. Quantitative results would depend on the airplane model and the ventilation system employed.

It is demonstrated in the present work that in all three models considered, aerosol in the 1 *μ*m–20 *μ*m size range is concentrated within one row of the index patient, and virtually, no particles make it past two rows from the index patient. Larger particles such as 50 *μ*m particles are practically only present in the row of the index patient. This localization of aerosol particles within one row of the index patient is in qualitative agreement with the experimental results of the extensive USTRANSCOM and AMC study on aerosol transport on Boeing 777–200 and 767–300.^11^ The comparison conducted between the different intervention measures reveals that using sneeze shields/guards between passengers on full capacity flights is nearly equivalent to vacating the middle seats when aggregate values are considered. While using sneeze guards on a full capacity flight was found to reduce the average deposition per passenger by 30%–35% and inhalable fraction per passenger by up to 50% relative to the reduced capacity model, it is more useful to consider aggregate values to account for the expected number of patients in the cabin zone. A reduced capacity flight would have fewer expected patients than a full capacity flight proportional to the ratio of the number of passengers. Aggregate values offer a relative indicator of risk to passengers, which accounts for a number of expected patients on the flight when comparing different intervention measures.

Using sneeze guards in full capacity flights may be more economically attractive than reducing passenger capacity through vacating middle seats. Based on the results of the present investigation, the following conclusions and recommendations are made:

(a) Reducing passenger capacity and using sneeze shields/guards on full capacity flights are shown to be nearly equivalent measures in terms of infectious aerosol transmission risk to passengers. Both measures eliminate the direct transmission of 50 *μ*m particles through inhalation and reduce the transmission of smaller particles (1–20 *μ*m) compared to full capacity flights with no shields.
(b) Experimental study of sneeze shields/guards should be conducted to further investigate their effects on airflow in the cabin especially in relation to passenger comfort and noise generation.
(c) Seats and walls are highly contaminated with particles even more than the ground. We recommend covering seats and disinfecting walls between flights.
(d) It was identified in the present work that particles take 2–3 min to deposit or leave the system as air in the cabin is rapidly renewed. Other studies have recommended loading passengers in small groups to reduce transmission.^11^ This is reasonable as aerosol transmission was shown to be localized with one row of the index patient. Loading passengers in the back seats first and waiting 2–3 min between each passenger group would allow particles to settle or leave the system before other passengers enter.

The present study is concerned with relative comparisons of different models and intervention measures considered. No conclusions can be made on the expected number of infections in any of the models, which necessitates characterization of the probability density functions of the viral shedding rates and the infectious dose of SARS-CoV2. Recommendations for the use of sneeze shields/guards are based on comparison with the widely employed intervention measure of reducing passenger capacity through vacating middle seats. The present study does not consider potentially increased time to deplane in the case of an emergency or other safety issues that may follow from the use of sneeze guards.

## AUTHORS' CONTRIBUTIONS

K.T. and M.A. made equal contribution to the study. O.M. created and refined the geometry.

## Data Availability

The data that support the findings of this study are available within the article.
